# Response of N_2_O emission and denitrification genes to different inorganic and organic amendments

**DOI:** 10.1038/s41598-022-07753-9

**Published:** 2022-03-10

**Authors:** Yajun Yang, Hexiang Liu, Jialong Lv

**Affiliations:** 1 College of Natural Resources and Environment, Northwest A&F University, Yangling, 712100 Shaanxi People’s Republic of China; 2 Key Laboratory of Plant Nutrition and the Agri-Environment in Northwest China, Ministry of Agriculture, Yangling, People’s Republic of China

**Keywords:** Ecology, Environmental sciences

## Abstract

Denitrification is a key biochemical process in nitrogen cycling and nitrous oxide (N_2_O) production. In this study, the impacts of different inorganic and organic amendments (OAs) on the abundance of denitrifying genes (*nirS*, *nirK* and *nosZ*) and the level of N_2_O emission were examined with incubation experiments. Six treatments included the indicated applications: (i) no fertilization (CK); (ii) urea application alone (U); (iii) wheat straw plus urea (U + WS); (iv) pig manure plus urea (U + PM); (v) compost product plus urea (U + CP); and (vi) improved compost product plus urea (U + IC). The results indicated that all fertilization treatments increased accumulative N_2_O emissions compared with the CK treatment. The U + WS, U + PM and U + CP treatments increased N_2_O emissions by 2.12–141.3%, and the U + IC treatment decreased N_2_O emissions by 23.24% relative to the U treatment. *nirK* was the dominant denitrification gene rather than *nirS* and *nosZ* found in soil. Additionally, the highest abundance of *nirK* gene was that with the U + PM treatment, and the lowest was that with the U + IC treatment. Additionally, changes in the *nirK* gene were highly correlated with levels of dissolved organic carbon (DOC), dissolved organic nitrogen (DON) and nitrate nitrogen (NO_3_^–^N). Automatic linear modeling revealed that N_2_O emission was closely related to the *nirK* gene, DOC and NO_3_^–^N. Overall, the use of urea and improved compost as co-amendments retarded N_2_O emission to a considerable degree compared with other OA additions.

## Introduction

Nitrous oxide (N_2_O) is one of the most prevalent greenhouse gases, and its warming potential is approximately 265 times that of CO_2_ on the 100-year scale^1^. In addition, N_2_O is an important substance that destroys the ozone layer^2^. Annual average N_2_O emissions from global cultivated fields total 6.4 × 10^12^ g [calculated as nitrogen (N)], which accounts for 25% of total global N_2_O emissions^3^. Agricultural fertilization management is the main source of N_2_O emissions. Application of nitrogen fertilizer stimulates N_2_O emission from cultivated soils^4,5^. Therefore, it is urgent to find an effective strategy for decreasing N_2_O emissions.

N_2_O is produced mainly through biological and abiotic processes. Denitrification is one of the main ways N_2_O is produced under microbe-driven conditions in soil^6^. Denitrification is a process in which nitrate (NO_3_^–^N) is sequentially reduced to nitrite (NO_2_^–^N), nitric oxide (NO), nitrous oxide (N_2_O), and finally dinitrogen gas (N_2_)^7^. It is essentially the inverse process of nitrification. Nitrite-reducing bacteria are encoded by nitrite reductase genes (*nirK* and *nirS*). In addition, reduction of N_2_O to N_2_ is one of the key steps in the whole process, and is encoded by the *nosZ* gene^8,9^. Nos-catalyzed N_2_O reduction plays a significant role in N_2_O emissions. Therefore, the abundance of *nirS*, *nirK* and *nosZ* denitrifying genes was used to study soil nitrogen cycling and N_2_O emissions^10,11^. At present, nitrogen addition affecting N_2_O emissions has attracted wide concern^12,13^. Wang et al.^14^ reported that nitrogen addition at 250 kg ha^−1^ promoted denitrification, thus contributing to comparable stimulation of N_2_O emissions. Albanito et al.^15^ proposed a linear relationship between N_2_O emissions and amounts of nitrogen fertilizer applied in tropical and subtropical regions. To reduce N_2_O emissions, combined application of organic materials and inorganic fertilizer has been endorsed by some scholars. The addition of organic material altered the abundance of denitrifiers and thus influenced the denitrification process^16,17^. Wang et al.^18^ found that organic materials stimulated the activity of the *nosZ* gene and decreased N_2_O emissions. However, some studies have suggested that organic materials (i.e., wastewater sludge, manure and vermicompost) provide carbon sources for denitrifiers and accelerate denitrification, thus increasing N_2_O emissions^11,19^. Although many studies have been focused on combined application of organic and inorganic fertilizers and its effect on N_2_O emissions in agricultural soils, the research conclusions are not yet consistent. Therefore, it is necessary to carry out research to determine the drivers of N_2_O emissions and propose reasonable fertilization strategies.

In addition, the identification of major denitrification-driving genes plays an important role in predicting N_2_O emissions. Yin et al.^20^ indicated that denitrifiers were more sensitive to inorganic fertilization than to organic fertilization. Harter et al.^21^ revealed that the *nosZ* gene is the decisive factor for N_2_O emissions. Hai et al*.*^22^ found that organic fertilizers increased the abundance of *nirS* and *nirK* genes and enhanced N_2_O emissions. Huang et al.^23^ reported that co-addition of urea and cattle manure decreased the abundance of *nirS* and *nirK* but increased N_2_O emissions. In addition, Xu et al.^24^ suggested that *nirS*-type denitrifying genes showed strong correlations with significant increases in N_2_O emissions from soils undergoing organic fertilization. Henderson et al.^25^ questioned whether the relationship between N_2_O emissions and the abundance of *nirS* and *nosZ* genes was significant. Other studies have focused on the impacts of co-amendment with inorganic and organic fertilizers on the abundance of denitrification bacterial communities and changes in soil properties^21,26–28^. However, there was still a knowledge gap regarding the dominant drivers of N_2_O emissions, including denitrifiers and environmental factors.

Therefore, this study was focused on the impacts of co-additions of urea and four organic additions (OAs), including wheat straw, pig manure, compost products and improved compost products, on N_2_O emissions. Additionally, fluorescence quantitative PCR and 16S rDNA sequencing were used to study changes in the abundance of denitrifying genes in soil caused by OAs and clarify the mechanism for the response of denitrifying gene abundance to added OAs. Particularly, the purposes of this research were (i) to compare the impacts of different OAs with urea on changes in N_2_O emissions; (ii) to understand the abundance of denitrifiers affected by different OAs; and (iii) to identify the relationships among N_2_O emissions, soil properties and the abundance of denitrifiers.

## Materials and methods

### Experimental soil collection

The tested soil was collected from the experimental station around Northwest A&F University in Shaanxi Province, China. The tested plot lies in a typical semiarid region with average annual precipitation of 533–631 mm. The basic soil properties before the experiment were as follows: pH = 7.53, soil organic carbon (SOC) = 0.79%, total nitrogen (TN) = 0.075%.

### Experimental materials collection

The four OAs included compost product (CP), wheat straw (WS), pig manure (PM) and improved-compost product (IC). PM and WS were collected from the farm and field of the experimental station. CP and IC products were produced in the greenhouse on the campus. CP was manufactured with PM and WS. IC was produced by the addition of bean dregs and biochar to the compost product. Previous studies confirmed that IC products increased SOC and TN contents more than compost products^29,30^. All OA materials were dried at 55 °C and then sieved through a 2 mm mesh. Basic physicochemical properties for all OAs are shown in the Supplementary Material.

### Experimental design

Six incubation experiments, including no fertilization (CK), urea N alone (U), 70% urea N plus 30% N from PM (U + PM), 70% urea N plus 30% N from WS (U + WS), 70% urea N plus 30% N from CP (U + CP) and 70% urea N plus 30% N from IC (U + IC), were conducted with three replicates to understand the responses of N_2_O emissions and denitrification genes to different fertilization practices. Fresh soil samples were first weighed (700 g for dry weight basis) and placed into plastic bottles. A total of 0.5 mg N g^−1^ of dry soil was added to each treatment. The amounts of urea and OAs were calculated by their total nitrogen content. The content of soil moisture was controlled at 60% of the water-holding capacity. Incubation was performed in a 25 °C environment for 77 days. Each plastic bottle was weighed, and distilled water was added to maintain constant moisture in the soil. All soil samples were divided into three parts after incubation: one was used for determination of soil properties after drying, one was used for measurements of NO_3_^–^N and NH_4_^+^-N at 4 °C, and the other was used for DNA determination at − 80 °C.

### Properties determination

Soil NO_3_^–^N and NH_4_^+^-N were determined with an AA3 flow analyzer by extraction with 2 mol L^−1^ KCl. Soil organic carbon (SOC) was measured through K_2_Cr_2_O_7_ digestion. Total nitrogen (TN) was measured by a Kjeldahl nitrogen analyzer^31^. In addition, soil moisture content was determined by weighing after drying. In addition, the soil microbial biomass carbon (MBC) and microbial biomass nitrogen (MBN) contents were determined using the chloroform fumigation-extraction method^32,33^. Briefly, triplicate soil samples were weighed and fumigated with ethanol-free chloroform for 24 h at 25 °C. The three fumigated and unfumigated samples from each treatment were extracted with 100 mL of 0.5 mol L^−1^ K_2_SO_4_ on a shaker at 220 rpm for 30 min and then filtered and determined with a TOC/TN analyzer. Dissolved organic carbon (DOC) was determined by a TOC-analyzer after extraction and filtering^34^. Dissolved organic nitrogen (DON) was measured using a flow analyzer after extraction with H_2_O^35^. N_2_O samples were collected on days 0, 3, 7, 14, 21, 28, 35, 49, 63 and 77 during incubation. All bottles were sealed for 10 min before sampling to ensure concentration of N_2_O emissions. N_2_O samples were determined by gas chromatography. The N_2_O fluxes and cumulative N_2_O emissions were calculated as described by Huang et al*.*^36^. The specific calculation formula is as follows:

$$ F = \frac{{\Delta {{C}}}}{\Delta t} \cdot  \frac{V}{m} \cdot  \frac{M}{\textit{22.4}} \cdot  \frac{\textit{273}}{{\textit{273} + T}} $$

*F* is the N_2_O fluxes (μg/kg/day), ∆C/∆t is the variation of concentration per unit time (μg/kg/day), V is the volume of the device (L), m is the dry soil weight (g), M/22.4 is the mass density of standard gases (g/L), and T is the incubation temperature (°C).

Cumulative N_2_O emission is the accumulation of the N_2_O fluxes during the incubation period (μg/kg).

### Microbial analyses

Soil DNA was extracted from 0.5 g of fresh soil using a DNA kit (Omega, GA, USA). The DNA solution extracted from each sample was studies with 1% agarose gel electrophoresis. The *nirK*, *nirS* and *nosZ* denitrifying genes were amplified by PCR using primers (Supplementary Materials). The abundances of denitrifiers were quantified by quantitative real-time PCR (qPCR) on an Illumina MiSeq platform in Beijing.

### Data analyses

Figures were prepared with Origin 2018. Significant differences among the treatments were analyzed by Duncan's multiple range test using IBM SPSS Statistics (version 20.0). Automatic linear modeling was conducted using IBM SPSS Statistics (version 20.0). Redundancy analyses (RDA) and principal component analyses (PCA) were conducted with Canoco (version 4.5).

## Results

### Changes in N_2_O emission

**Figure 1:**
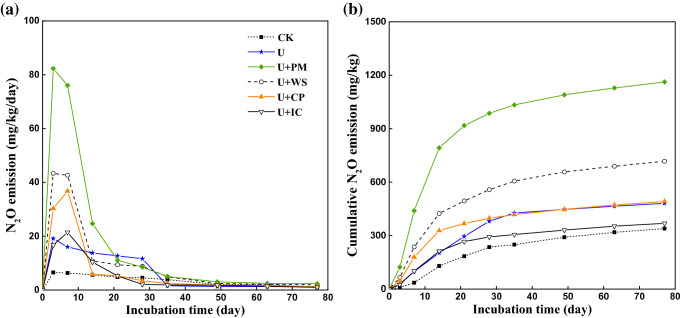
The changes of N_2_O emission (**a**) and cumulative N_2_O emission (**b**) during the incubation affected by different OAs. *CK* no chemical fertilizer, *U* urea, *U* + *WS* urea plus wheat straw, *U* + *PM* urea plus pig manure, *U* + *CP* urea plus compost, *U* + *IC* urea plus improved compost.

N_2_O emissions influenced significantly by different OAs are shown in Fig. 1a. A similar trend for N_2_O emissions among all treatments was observed. N_2_O emissions increased within the first 7 days and declined thereafter. N_2_O emissions peaked on day 3 for the CK, U, U + WS and U + PM treatments and peaked on day 4 for the U + IC and U + CP treatments. The OAs increased N_2_O emissions during the whole incubation process compared with the CK treatment. The maximum peak value for N_2_O emissions among all OAs was that seen with the U + PM treatment (76.04 mg/kg/day), and the minimum value of N_2_O emissions was that seen with the U + IC treatment (21.44 mg/kg/day). Compared with other OA treatments, compost amendments (U + IC, U + CP) decreased the peak values of N_2_O emissions. Furthermore, the highest cumulative N_2_O emissions were seen with the U + PM treatment, which were 241.8% higher than the levels seen with the CK sample; this was followed by the U + WS (111.1%), U + CP (44.63%), U (41.61%) and U + IC (8.70%) treatments (Fig. 1b). In addition, cumulative N_2_O emissions for the U + WS, U + PM and U + CP treatments were 49.05%, 141.3% and 2.12% higher relative to the U treatment, but the cumulative N_2_O emission for the U + IC treatment was 23.24% lower compared with that of the U treatment. These results indicated that relative to application of urea alone, mixing urea with wheat straw, pig manure and compost products promoted N_2_O emissions during incubation. In addition, IC retarded N_2_O emissions to a greater degree compared with other OA additions.

### Changes in soil properties

The basic properties of soils are shown in Table 1. The highest SOC content was observed for the U + WS treatment, followed by the U + IC and U + CP treatments, and these levels were significantly higher than that of the CK treatment (P < 0.05). However, there was no significant difference in SOC contents for the U + PM and CK treatments (P > 0.05). Relative to the CK treatment, OAs significantly increased the levels of DON, NH_4_^+^-N and NO_3_^–^N. The highest NO_3_^–^N, NH_4_^+^-N and DON contents were found for the U + PM treatment, and these were higher than those seen for other OA treatments (P < 0.05). However, the DOC contents with all OA treatments except for U + IC differed significantly from that of the CK treatment (P < 0.05). The maximum DOC concentration was observed for the U + WS treatment. In addition, only the U + PM treatment, among all OA treatments, failed to increase the MBN content significantly (P > 0.05). The U treatment did not significantly increase any soil properties during incubation relative to the CK treatment except for NO_3_^–^N (P > 0.05).

**Table 1:** Soil properties in different treatments with OAs.

Treatments	SOC (g/kg)	NH_4_^+^-N (mg/kg)	NO_3_^–^N (mg/kg)	MBN (mg/kg)	DON (mg/kg)	DOC (mg/kg)
CK	6.53 ± 0.85a	0.58 ± 0.01a	49.35 ± 10.6a	0.48 ± 0.24a	7.14 ± 3.06a	150 ± 48a
U	6.59 ± 0.80a	0.63 ± 0.01a	80.60 ± 11.2b	0.53 ± 0.18a	7.21 ± 3.01a	155 ± 44a
U + PM	6.91 ± 0.58a	0.37 ± 0.02b	219.4 ± 16.94c	0.85 ± 0.15a	60.53 ± 4.19b	290 ± 22b
U + WS	8.88 ± 0.32b	0.13 ± 0.07c	147.1 ± 10.05d	1.49 ± 0.26b	54.22 ± 3.08c	330 ± 32b
U + CP	8.13 ± 0.65b	0.18 ± 0.03c	182.6 ± 16.25e	0.97 ± 0.15ac	54.79 ± 1.58bc	210 ± 18c
U + IC	8.63 ± 0.55b	0.25 ± 0.02d	178.1 ± 10.45e	0.94 ± 0.25c	48.14 ± 4.22d	180 ± 9.1ac

*CK* no urea and OAs, *U* urea, *U* + *WS* urea plus wheat straw, *U* + *PM* urea plus pig manure, *U* + *CP* urea plus compost, *U* + *IC* urea plus improved compost.

Values are mean ± standard deviation (n = 3). The different letters indicate significant differences at the 0.05 probability level.

### Abundances of *nirK*, *nirS *and *nosZ* denitrifying genes

**Figure 2:**
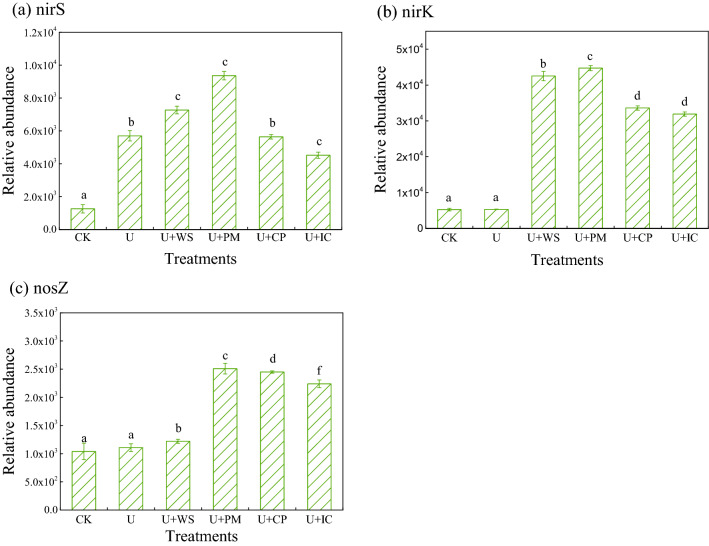
Relative abundance of denitrification genes during the incubation affected by different OAs. Error bars indicate standard deviations (n = 3). The different letters indicate significant differences (P < 0.05) (nirS (**a**); nirK (**b**); nosZ (**c**); *CK* no urea and OAs, *U* urea, *U* + *WS* urea plus wheat straw, *U* + *PM* urea plus pig manure, *U* + *CP* urea plus compost, *U* + *IC* urea plus improved compost).

The relative abundances of denitrification genes (*nirK*, *nirS* and *nosZ*) were determined during incubation for all treatments (Fig. 2). The copy numbers for *nirK*, *nirS* and *nosZ* varied within the ranges 5.26 × 10^3^–4.47 × 10^4^, 1.26 × 10^3^–9.36 × 10^3^ and 1.04 × 10^3^–2.51 × 10^3^, respectively. The *nirK*-type denitrification genes exhibited the highest abundance after incubation. Compared with the CK treatment, all OA treatments (U + CP, U + IC, U + PM and U + WS) significantly increased the abundance of the *nirK* gene. The highest copy number of the *nirK* gene was seen for the U + PM treatment, followed by the U + WS, U + CP and U + IC treatments. In addition, there were no significant differences between the U + IC and U + CP treatments. However, the *nosZ* gene had the lowest abundance after incubation. Compared with the CK treatment, all OA treatments except the U + WS treatment significantly increased the copy number of the *nosZ* gene. The maximum abundance of the *nosZ* gene was observed for the U + PM treatment. There was no significant difference between the CK and U treatments. For abundance of the *nirS* gene, the U and OA treatments all significantly increased the abundance relative to the CK treatment. The highest *nirS* gene abundance was seen for the U + PM treatment, followed by the U + WS, U + CP and U + IC treatments. However, there was no significant difference between the U and U + CP treatments. Among all denitrification genes, the U treatment significantly increased only the abundance of *nirS* genes. These results indicated that co-additions of urea and OAs increased the abundance of *nirK* genes more, while urea alone increased the abundance of *nirS* genes more.

### Correlations of denitrifying gene abundance and soil properties

**Figure 3:**
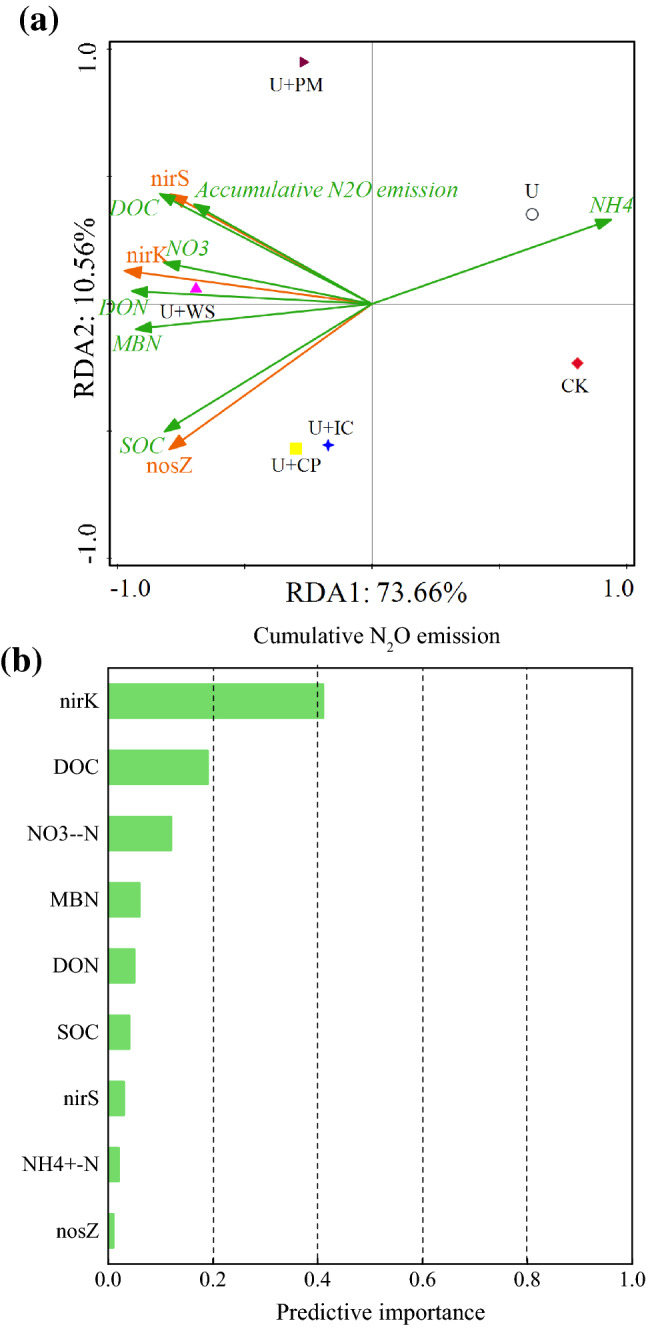
Principal component analysis (PCA) of soil properties, cumulative N_2_O emission and abundance of denitrification genes (**a**); predictive importance of denitrifier gens abundance, and soil properties on cumulative N_2_O emission as determined by automatic linear modeling (**b**) (*DOC* dissolved organic carbon, *MBN* microbial biomass carbon, *DON* dissolved organic nitrogen, *SOC* soil organic carbon, *CK* no urea and OAs, *U* urea, *U* + *WS* urea plus wheat straw, *U* + *PM* urea plus pig manure, *U* + *CP* urea plus compost, *U* + *IC* urea plus improved compost).

PCA was used to analyze the abundances of denitrifiers among all treatments (Fig. 3a). The results showed that the explanations of Axis 1 and Axis 2 contributed 73.66% and 10.56%, respectively, to changes in denitrifying genes (*nirS*, *nirK* and *nosZ*). The OA treatments were largely separated from the CK and U treatments along PCA1. Additionally, the U + PM and U + WS treatments were segregated from the U + CP and U + IC treatments along PCA2. The results suggested that OA application significantly influenced the abundance of the three types of denitrifiers, and OAs had stronger effects on the abundance of denitrifiers than urea application alone. Correlations among soil properties, accumulative N_2_O emissions and denitrifying gene abundance showed that the abundance of the *nirS* gene was significantly associated with accumulative N_2_O emissions (r = 0.876, P < 0.05) and DOC (r = 0.859, P < 0.05). The abundance of the *nirK* gene was correlated with DON (r = 0.977, P < 0.1), NO_3_^–^N (r = 0.880, P < 0.05), DOC (r = 0.865, P < 0.05), MBN (r = 0.852, P < 0.05) and NH_4_^+^-N (r = − 0.880, P < 0.05). In addition, the abundance of the *nosZ* gene was mainly related to SOC (r = 0.965, P < 0.1), MBN (r = 0.836, P < 0.05) and NH_4_^+^-N (r = − 0.931, P < 0.1). In addition, the results of automatic linear modeling showed that *nirK* abundance, DOC, NO_3_^–^N, MBN and DON were the main factors influencing N_2_O emissions (Fig. 3b).

## Discussion

Different fertilization practices influenced N_2_O emissions from soil. Soil N_2_O emissions peaked on day 3 for the CK, U, U + WS and U + PM treatments, while those for the U + CP and U + IC treatments peaked on day 7. This may be attributed to the fact that WS and PM provided more rapidly available N than CP and IC^37^. In addition, compared with urea application alone, the U + CP, U + WS and U + PM treatments increased cumulative N_2_O emissions by 2.13–49.06%, which was within the increase range of 27–74% seen with pig slurry and compost amendments^38^. However, the U + IC treatment decreased cumulative N_2_O emissions by 23.24% relative to urea alone. Therefore, the combined application of IC and urea reduced N_2_O emissions in soil because IC was more stable than CP, PM and WS. Additionally, OA treatment increased cumulative N_2_O emissions during incubation relative to the CK treatment. This might be because OAs provided more substrates for denitrification through mineralization. Denitrification could result in a rapid reduction in NO_3_^–^N content and promote the emission of N_2_O. Previous studies showed that the NO_3_^–^N concentration was an important factor affecting the denitrification rate and N_2_O release^39–43^. Increasing the concentration of NO_3_^–^N could significantly increase N_2_O release. Compared with urea application alone, the OA additions increased the content of NO_3_^–^N in soil; furthermore, PM application provided more NO_3_^–^N than WS, CP and IC. Therefore, PM application promoted changes in N_2_O emissions, which was consistent with the variations in NO_3_^–^N. In addition, SOC increased by addition of OAs to the soil. The importance of SOC as a factor affecting denitrification and N_2_O emissions has been reported by Chen et al*.*^44^. However, in this study, the effect of SOC on denitrifiers and N_2_O emissions was lower than that of DOC due to the narrow range of SOC changes occurring in a short-term incubation experiment. Carbon availability was the key controlling factor for denitrification in soil^45^. Compared with the U + PM treatment, the U + WS treatment produced a higher DOC content but lower N_2_O emissions, which may be due to the higher C/N ratio in WS. Microbial fixation of carbon in the straw treatment limited carbon effectiveness. In addition, of all treatments involving addition of organic material, the modified compost addition treatment produced the lowest DOC content due to its higher degree of humification, which reduced the abundance of *nirK* genes and therefore reduced N_2_O emissions from the soil^37^.

N_2_O emissions were highly related to soil physiochemical properties and the abundance of denitrifiers, which was consistent with the findings of Sun et al*.*^46^. Denitrifying bacteria are active in soil biological denitrification^11,47^. Many studies showed that fertilization increased the number of denitrifying microorganisms in the soil by providing substrates and energy for denitrifying bacteria and promoting their growth and reproduction^37,46,48^. In this study, the OA addition increased the abundance of denitrifying genes in soil. The contributions of *nirK* to N_2_O emissions were higher than those of *nirS* and *nosZ*. In addition, application of pig manure significantly increased *nirK* gene abundance and thus promoted N_2_O emissions more than other OAs. Yoshida et al*.*^49^ also found that application of organic manure increased the abundance of *nirK* genes in rice paddy soil more than that of *nirS* genes. However, there are some studies showing the opposite results. For example, Yin et al*.*^50^ found that organic manure changed the abundance of the *nirS* gene community in black soil during long-term treatment but not that of *nirK*. Barrett et al*.*^51^ confirmed that a higher abundance of *nirS*-type genes was observed in carbon- amended soil relative to other genes. This may be related to the different sources and nature of available carbon in amended soil. Environmental factors significantly influenced the abundance of denitrifiers in this study^52,53^. Positive correlations were observed between NO_3_^–^N, DON, DOC and the abundance of the *nirK* gene. These findings suggested that NO_3_^–^N, DON and DOC levels were important factors affecting the abundance of the soil denitrifying bacterial community, thus affecting denitrification with different OA applications. This may be due to differences in the availability of C and N in OA-amended soil.

## Conclusion

Fertilization treatments increase denitrification and N_2_O emissions relative to the CK treatment. In addition, compared with urea alone, combined application of pig manure and urea provided more available N (DON and NO_3_^–^N) and increased the abundance of *nirK* genes, thus increasing cumulative N_2_O emissions from the soil. However, combined applications of improved compost products and urea reduced accumulated N_2_O emissions by decreasing the abundance of *nirK* genes. Overall, the different fertilization practices affected the abundance of denitrifiers, denitrification and soil properties. From the perspective of soil N_2_O emission reduction, we recommend the application of improved compost products and urea to the soil.

## Supplementary Information

Supplementary Information.

## Data Availability

All data generated or analysed during this study are included in this published article (and its Supplementary Information files).
