# Supplementary material for: Response of N2O emission and denitrification genes to different inorganic and organic amendments
Source: Sci Rep. 2022 Mar 10;12:3940. doi: 10.1038/s41598-022-07753-9 (PMC8913736; doi:10.1038/s41598-022-07753-9)
Supplement: Supplementary file 1 — Supplementary Information. [file 41598_2022_7753_MOESM1_ESM.doc]

**SUPPORTING INFORMATION**

**Response of N_2_O emission and denitrification genes**

**to different inorganic and organic amendments**

Yajun Yang^1,2^, Hexiang Liu^1,2^, Jialong Lv^1,2^,*

*^a^College of Natural Resources and Environment, Northwest A&F University, Yangling, Shaanxi Province 712100, PR China*

*^b^Key Laboratory of Plant Nutrition and the Agri-environment in Northwest China, Ministry of Agriculture, China*

**Materials and Methods**

***Experimental materials collection***

**Table S1**

Chemical properties of organic amendments used in 97-day incubation experiment

| Material | C% (DM) | N% (DM) | C/N ratio | pH |
| --- | --- | --- | --- | --- |
| PM | 31.55(0.14) | 2.44(0.02) | 12.93 | 6.56(0.06) |
| WS | 40.66(0.12) | 1.09(0.03) | 37.34 | 8.21(0.01) |
| CP | 27.25(0.09) | 2.09(0.01) | 13.03 | 7.79(0.08) |
| IC | 32.19(0.05) | 2.45(0.33) | 13.32 | 8.15(0.07) |

Notes: n.d.=not determined, C=carbon, N=nitrogen, DM=dry matter, PM=pig manure, WS=wheat straw, CP=compost, IC=improved compost.

***Data determination and analyses***

***Microbial analyses***

**Table S2.** The primer sequences for each target gene used in this study.

| Gene name | Primer |
| --- | --- |
| *nirS* | F: GTSAACGTSAAGGARACSGG  R: GASTTCGGRTGSGTCTTGA |
| *nirK* | F: ATCATGGTSCTGCCGCG  R: GCCTCGATCAGRTTGTGGTT |
| *nosZ* | F: CGYTGTTCMTCGACAGCCAG  R: CGSACCTTSTTGCCSTYGCG |

**S. The details of qPCR reaction system and amplification conditions**

The qPCR reaction system comprised 1 μL DNA template, 0.5 μL 10 μM forward and reverse primers (Beijing Auwigene Tech, Ltd, China), 12.5 μL 2×MltraSYBR Mixture, and 10.5 μL of sterilized double distilled H_2_O.

The qPCR amplification reaction was as follows: (1) initial denaturation for 5 min at 94°C; (2) 40 cycles at: 94°C for 30 s, annealing for 30 s at 60°C, and then extension at 72°C for 1 min. In order to eliminate the effects of PCR inhibitors on qPCR in the DNA extracts, the DNA template used for qPCR was diluted 10 times. The presence of inhibitory compounds in the extracted DNA was checked by qPCR using serially diluted samples. Melting curve analysis was used to detect nonspecific amplification. Each gene was quantified in triplicate using a standard curve and a negative control. The gene copy numbers in the samples were calculated using the external standard curve method. The abundances of genes were calculated as: copy number of gene/copy number of 16S rDNA.
